# Risk factors for recurrent injuries in victims of suspected non-accidental trauma: a retrospective cohort study

**DOI:** 10.1186/1471-2431-14-217

**Published:** 2014-08-31

**Authors:** Katherine J Deans, Jonathan Thackeray, Jonathan I Groner, Jennifer N Cooper, Peter C Minneci

**Affiliations:** 1 Center for Surgical Outcomes Research and Center for Innovation in Pediatric Practice, The Research Institute at Nationwide Children’s Hospital, 700 Childrens Drive, JWest - 4th floor, Columbus, OH 43205, USA; 2 Department of Surgery, Nationwide Children’s Hospital, Columbus, OH, USA; 3 Division of Child and Family Advocacy, Nationwide Children’s Hospital, Columbus, OH, USA

**Keywords:** Non-accidental trauma, Child abuse, Injury, Recurrence

## Abstract

**Background:**

Many children who are victims of non-accidental trauma (NAT) may be repeatedly evaluated for injuries related to maltreatment. The purpose of this study was to identify risk factors for repeated injuries in children with suspected NAT.

**Methods:**

We conducted a retrospective cohort study using claims data from a pediatric Medicaid accountable care organization. Children with birth claims and at least one non-birth related claim indicating a diagnosis of NAT or skeletal survey in 2007–2011 were included. Recurrent events were defined as independent episodes of care involving an urgent/emergent care setting that included a diagnosis code specific for child abuse, a CPT code for a skeletal survey, or a diagnosis code for an injury suspicious for abuse. Cox proportional hazards models were used to examine risk factors for recurrent events.

**Results:**

Of the 1,361 children with suspected NAT, a recurrent NAT event occurred in 26% within 1 year and 40% within 2 years of their initial event. Independent risk factors for a recurrent NAT event included a rural residence, age < 30 months old, having only 1 or 2 initially detected injuries, and having a dislocation, open wound, or superficial injury at the previous event (p ≤ 0.01 for all).

**Conclusions:**

Over 25% of children who experienced a suspected NAT event had a recurrent episode within one year. These children were younger and more likely to present with “minor” injuries at their previous event.

## Background

Non-accidental trauma (NAT) is a leading cause of injury and death throughout early childhood [1,2]. Repeated evaluations in the medical setting for traumatic injuries should raise concerns that these injuries may be caused by either negligent behavior on the part of the caretaker or by recurrent intentional mechanisms.

Rates of recurrent non-accidental traumatic injuries have been reported to be as high as 30-50%, and are associated with increased morbidity and mortality [3-8]. Previously reported predictors of recurrent NAT include prior child protective services involvement, history of domestic violence, chronicity of maltreatment, child’s age, parental history of maltreatment as a child, and parental substance abuse, criminal record, and mental health issues, or after specific injuries [5,6,9-13]. These previous studies are limited in that they either do not assess risk factors related specifically to trauma, such as sentinel traumatic events, or they do not address recurrence of maltreatment. The purpose of this study was to identify patterns of injuries and factors associated with suspected episodes of recurrent NAT in a cohort of young children enrolled in a Medicaid managed care program who had at least one highly suspicious encounter for NAT.

## Methods

### Data source

Partners for Kids (PFK) is Nationwide Children’s Hospital’s pediatric accountable care organization. PFK contracts with the Medicaid Managed Care Organizations in Central and Southeastern Ohio to manage the care of almost 300,000 children across 37 counties, from urban Columbus to rural Appalachia. At the time of this study, over 2,000 physicians were submitting claims to PFK. The PFK claims database includes information on all billable medical care, procedures, and encounters for its enrollees, allowing for tracking of patients over time, across institutions, and across both inpatient and outpatient encounters. Access to this claims database is available to researchers at our institution, though is not freely available to individuals outside of our institution, and was granted by the PFK accountable care organization.

### Study population

**Figure 1:**
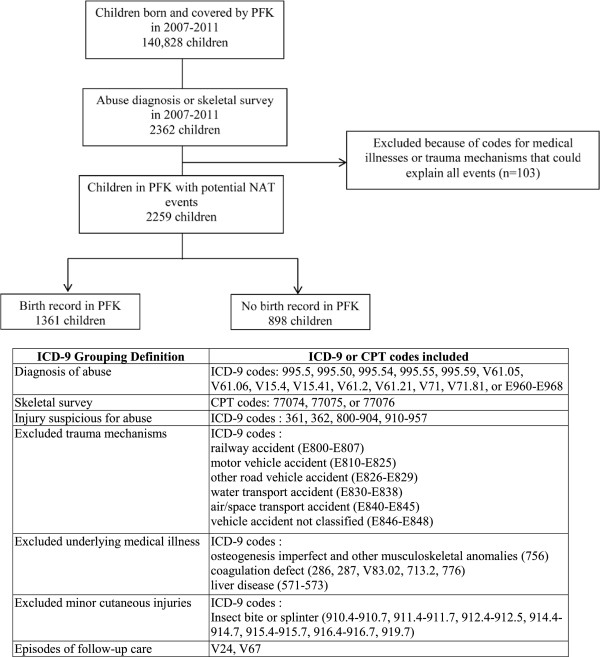
Determination of Study Population.

This study used enrollment data and facility and professional claims data from January 2007 to December 2011 for children born during this time period. We identified all children with a birth record claim who also had at least one claim indicating a diagnosis of abuse (physical, emotional, or neglect) or a skeletal survey at a non-birth related episode of care. (Figure 1) Suspected NAT events were defined as episodes of care in which a claim contained either (a) an International Classification of Diseases, Ninth Revision, Clinical Modification (ICD-9-CM) discharge diagnosis code specific for child abuse, (b) a Current Procedural Terminology (CPT) coded skeletal survey, or (c) ICD-9 coded injuries suspicious for abuse; these events could be the event that brought the child into the study cohort, or they could occur either before or after that event. We excluded events that had an ICD-9 E-code for a trauma mechanism that could explain the injury or an ICD-9 code for an underlying medical illness that could explain the injury or need for skeletal survey. Episodes of care coded as follow-up care were excluded. Episodes of care with only a diagnosis of minor cutaneous injury from a specific mechanism and no other codes indicative of suspected NAT were also excluded. In order to include all claims for care related to a single incident of suspected NAT, an episode of care encompassed all claims for service provided concurrently or within two days of the care documented in the claim. In order to minimize the risk of defining claims for follow-up care as new events, only episodes of care that included encounters in the emergency department, urgent care, or inpatient setting were considered for inclusion as recurrent events. Figure 1 outlines cohort development and includes all ICD-9 and CPT codes used to define the cohort and events.

### Independent variables

Variables determined at the time of each event included age, sex, days since last event, the presence of symptoms or diseases of the respiratory system, digestive system, nervous system and sense organs, skin and subcutaneous tissue, endocrine, nutritional, metabolic, or immunity disorders, vaccination during the episode of care, location, type, and mechanism of injuries, number of injuries, injury severity (evaluated as the probability of death based on the trauma mortality prediction model, TMPM-ICD9) [14,15], and death during the episode of care. The type and mechanism of injury were defined using ICD-9 diagnosis codes and E-codes respectively. The location of each injury was categorized into one of six body regions based on the Abbreviated Injury Scale [16]. The number of injuries was defined as the number of unique injury diagnosis codes listed during the episode of care of the event. Because family socioeconomic status (SES) indicators were not available, zip code level SES variables (median household family income, percentage of the population over age 25 with a Bachelor’s degree or higher) and urban vs. rural residence were determined from the 2000 U.S. Census based on each child’s zip code at their first event [17]. Enrollment duration in months and enrollment continuity were determined for each child. Other independent variables were determined according to their presence on any claim submitted prior to the suspected NAT event including musculoskeletal disease, congenital anomalies, and prematurity.

### Statistical analysis

Characteristics at each suspected NAT event and in children with and without recurrent events were summarized using descriptive statistics (medians and inter-quartile ranges (IQR) or frequencies and percentages). Kaplan-Meier curves were used to display the proportion of children with recurrent events over time after the initial event. To determine risk factors for recurrent events, we used an extension of the Cox proportional hazards model for recurrent event data, the Prentice, Williams and Peterson gap time (PWP-GT) model [18]. Events beyond the fifth event were not included due to their insufficient number for analysis. Predictor variables in these models were the independent variables as measured at the previous suspected NAT event, with the exception of the zip code based variables, which were determined at the first event only. The reported hazard ratios (HR) estimate the relative hazard rates of having an event in those with and without the characteristic being examined.

For the examination of multivariable associations between the predictors and the time to the next suspected NAT event, Cox proportional hazards PWP-GT recurrent event models were used. All variables with bivariate associations significant at p < 0.20 were included with subsequent variable elimination until all remaining variables had p < 0.10. The final multivariable model revealed the overall associations of factors measured at any particular event with the risk of a subsequent suspected NAT event, after adjustment for other measured risk factors. We included all children regardless of their duration of follow-up in our analyses in order to minimize selection bias; in all of the survival analyses performed, children were included in the pool of patients at risk for subsequent events from the time of their initial event until the end of their last month of enrollment in PFK during the study period. Subsequently, several sensitivity analyses were performed to evaluate how the inclusion of patients with short follow-up, discontinuous follow-up, or without birth records in the database affected the results. The sensitivity analyses involved repeating the multivariable modeling excluding those children who did not maintain continuous enrollment in PFK, then excluding those children with less than 60 days of follow-up after their first event, and finally performing the analyses in those children who did not have birth records in the PFK database. All statistical analyses were performed using SAS (Statistical Analysis Software v9.3, Cary, NC). The conduct of this study was approved by Nationwide Children’s Hospital Institutional Research Board with a waiver of informed consent. This research study has adhered to the STROBE guidelines for observational studies as outlined at http://www.strobe-statement.org. Additional file 1.

## Results

### Identification of cohort

Of the 140,828 children born and enrolled in PFK from 2007–2011, 2,362 had a claim with either a diagnosis of child maltreatment or a skeletal survey. Sixty-one percent of these children (n = 1,434) had birth records in the PFK database. After removing events with diagnosis codes for a medical illness or trauma mechanism that could potentially explain the injuries, the cohort was further refined to 1,361 children who were included in the main analysis (Figure 1).

### Frequency of recidivism

**Figure 2:**
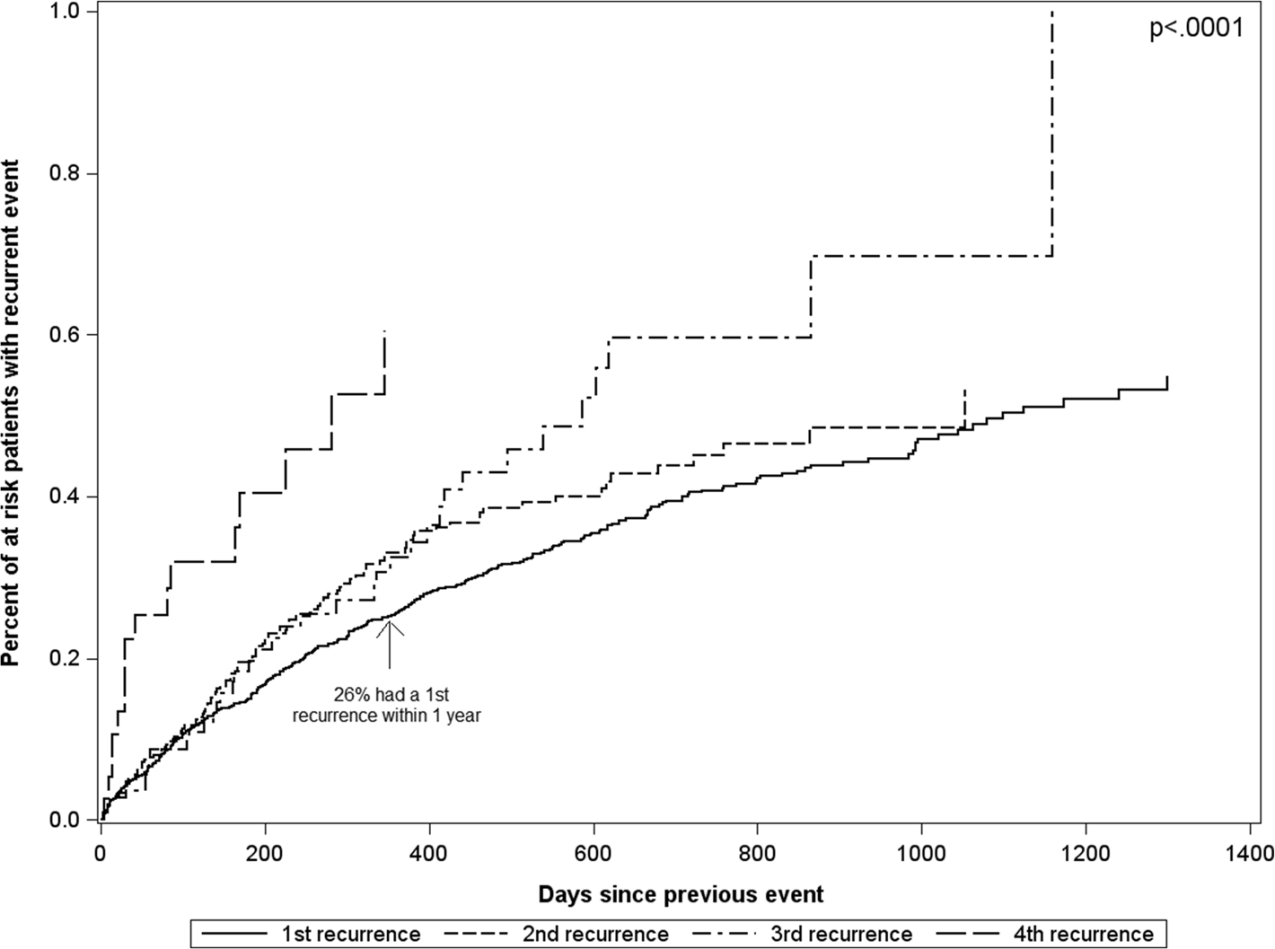
**Kaplan-Meier failure curves for time between recurrent events.** The percent of at risk patients that have a recurrent event (y-axis) over time since their previous event (x-axis) is displayed. For example, all patients with a first event are at risk for a 1^st^ recurrence (solid black line). At 1 year after their first event, 26% of these children have had a 1^st^ recurrence. The time between events significantly decreased with each increasing event number (p < 0.0001; derived from a Wald test of event number (modeled as an ordinal variable) in a Prentice, Williams and Peterson gap time (PWP-GT) Cox proportional hazards model for time between events).

Three hundred and seventy-three (27.4%) patients in our cohort had more than one episode of care for a suspected NAT event during the study period (Table 1). The incidence of suspected NAT events in the total cohort was 49 events per 1000 person-months. Two hundred and sixty-one children had 2 events, 74 children had 3 events, 22 children had 4 events, 13 children had 5 events, and 3 children had 6 events during the study period. Of all of these events, 51% had documentation of a skeletal survey, 35% had an abuse diagnosis, and 65% had an injury. Thirty percent of all events had only injuries, with no evidence of a skeletal survey or abuse diagnosis; at these events, the most common injuries were open wounds (32%) and contusions (27%). These potentially accidental injuries equated to an injury rate of 177/1000 person-years. Based on Kaplan-Meier analysis, 26% of the children had ≥1 recurrent event within 1 year of their initial event and 40% had ≥1 recurrence within 2 years of their initial event. The time between events decreased significantly with each subsequent event (Figure 2, p < 0.0001). It is important to note that the duration of follow-up after the initial event widely varied (median (IQR) 383 days (145, 773)). However, the finding of significantly decreased time between events with increasing event number held in the subsample of 476 children with at least 600 days of follow-up after their first event (p = 0.005).

**Table 1:** Demographics, comorbidities, and injury characteristics at first event in children with and without recurrent events

**Characteristic (Total N = 1361)**	**No suspected recurrent NAT events, (N = 988)**	**Suspected recurrent NAT events, (N = 373)**	**Hazard ratio**	**(95% CI)**		P***
Male	492 (49.8)	207 (55.5)	1.15	0.97	1.36	0.10
Lives in urban area*	760 (77.6)	266 (71.3)	0.71	0.59	0.86	**<0.001**
Age in months						
0-6	298 (30.2)	123 (33.0)	**ref**			**0.03**
6-12	187 (18.9)	81 (21.7)	0.88	0.68	1.15	
12-18	168 (17.0)	80 (21.5)	1.10	0.85	1.41	
18-24	120 (12.2)	44 (11.8)	1.06	0.79	1.41	
24-30	77 (7.8)	28 (7.5)	1.04	0.75	1.45	
>30	138 (14.0)	17 (4.6)	0.62	0.42	0.90	
Injury type, N (%)						
Fracture	234 (23.7)	81 (21.7)	0.903	0.722	1.13	0.37
Dislocation	19 (1.9)	15 (4.0)	1.751	1.17	2.621	**0.007**
Burn	49 (5.0)	21 (5.6)	0.985	0.676	1.435	0.94
Retinal hemorrhage	30 (3.0)	11 (3.0)	1.575	0.838	2.96	0.16
Intracranial	67 (6.8)	20 (5.4)	0.938	0.617	1.426	0.76
Abdominal thoracic	17 (1.7)	4 (1.1)	0.707	0.302	1.655	0.42
Open wound	59 (6.0)	45 (12.1)	1.564	1.243	1.968	**0.001**
Superficial Injuries	55 (5.6)	59 (15.8)	1.616	1.266	2.063	**<0.001**
Contusions	176 (17.8)	93 (24.9)	1.195	0.972	1.469	0.09
Spinal cord	6 (0.6)	1 (0.3)	0.899	0.223	3.614	0.88
Location of injury, N (%)						
Head/neck	192 (19.4)	83 (22.3)	0.996	0.806	1.232	0.97
Face	40 (4.1)	24 (6.4)	1.468	1.051	2.051	**0.02**
Chest	52 (5.3)	12 (3.2)	0.723	0.448	1.166	0.18
Abdomen and pelvic contents	40 (4.1)	9 (2.4)	0.748	0.452	1.24	0.26
Extremities or pelvic girdle	238 (24.1)	99 (26.5)	1.149	0.948	1.391	0.16
External	370 (37.5)	178 (47.7)	1.221	1.013	1.472	**0.04**
Mechanism of injury, N (%)						
Cut/pierce	4 (0.4)	2 (0.5)	1.335	0.673	2.648	0.41
Fall	160 (16.2)	63 (16.9)	1.238	0.992	1.543	0.06
Fire/Burn	35 (3.5)	12 (3.2)	0.958	0.618	1.485	0.85
Natural/environmental	9 (0.9)	3 (0.8)	0.829	0.361	1.906	0.66
Poisoning	14 (1.3)	3 (0.8)	0.95	0.33	2.733	0.92
Struck by/against	43 (4.4)	20 (5.4)	1.021	0.704	1.482	0.91
Number of injuries, N (%)						
0	387 (39.2)	86 (23.1)	**ref**			**<0.001**
1	182 (18.4)	115 (30.8)	1.803	1.435	2.265	
2	140 (14.2)	85 (22.8)	1.594	1.231	2.063	
3	63 (6.4)	31 (8.3)	1.378	0.953	1.992	
4	59 (6.0)	16 (4.3)	1.103	0.689	1.766	
≥5	157 (15.9)	40 (10.7)	1.161	0.841	1.601	
Injury Severity (TMPM-ICD9 probability of death)**, median (IQR)	0.046 (0.013, 0.107)	0.023 (0.010, 0.069)	0.478	0.104	2.194	0.34

Characteristics shown were determined at the first event. *Based on the child's zip code at their first event and on data from the 2000 Census. **TMPM-ICD9 = trauma mortality prediction model, Group without recurrent event(s): N = 502, Group with recurrent event(s): N = 193. ***P value in a univariable Cox proportional hazards Prentice, Williams and Peterson gap time (PWP-GT) model for time between events. The first four recurrent events were included in the models. Values of the risk factors at the previous event were the independent variables in this model, with the exception of the zip code based variables, which were determined only at the first event.

### Demographics, comorbidities and injury characteristics of patient population

Characteristics and injuries of the children with a single event were compared to children with recurrent events (Table 1). Among those children with multiple events during the time they remained in PFK, the median time between the first and second events was 191 days (IQR 69, 389). The median probability of death, according to the trauma mortality prediction model (TMPM-ICD9), was higher at the first event (3.1%) than at subsequent events (<2% for all).

### Risk of recidivism

Results of univariate comparisons of demographics, comorbidities and injuries between children with a single event and children with recurrent events are shown in Table 1. Factors independently associated with the risk of suspected recurrent NAT based on multivariable modeling are shown in Table 2. Living in a rural area and being less than 30 months of age at any event were associated with a higher risk of having a subsequent event (Table 2). Having a dislocation, open wound, or superficial injury (p ≤ 0.01 for all) was associated with an increased risk of having a subsequent event. In addition, children who had 1–2 injuries at any event were more likely to have a subsequent event, whereas children with 3 or more injuries were not at increased risk for another event compared to children with no injury diagnoses. The most common body locations of dislocations, open wounds, and superficial injuries at the initial event among children with suspected recurrent NAT events were as follows: 14 of 15 (93%) children with dislocations had elbow dislocations, 26 of 45 (58%) children with open wounds had open wounds of the face, nose, or mouth, and 28 of 59 (47%) children with superficial injuries had superficial injuries of the face, neck, or scalp.

**Table 2:** Multivariable Cox Proportional Hazards model for recurrent events

**Variable**	**Hazard ratio**	**95% CI**		**P**
Lives in a rural area (Non-MSA vs. MSA)*	1.37	1.14	1.67	0.001
Age ≤30 months vs. >30 months	1.67	1.20	2.33	0.002
Dislocation	1.77	1.15	2.72	0.01
Open wound	1.54	1.22	1.94	<0.001
Superficial injury	1.50	1.17	1.92	0.002
Number of injuries				
1-2 vs. 0 injuries	1.85	1.42	2.40	<0.001
≥ 3 vs. 0 injuries	1.03	0.74	1.43	0.86

Results are from a Cox proportional hazards Prentice, Williams and Peterson gap time (PWP-GT) model for time between events. Values of the risk factors at the previous event were the independent variables, with the exception of the zip code based variables, which were determined only at the first event. The global p-values for differences among age groups (0–6, 6–12, 12–18, 18–24, 24–30, and >30 months) and number of injuries (0, 1, 2, 3, 4, ≥5) were significant at p < 0.05, so these categories were collapsed into the smallest number of categories showing statistically significantly different associations with the outcome after adjustment for multiple comparisons using Fisher’s least significant difference test. *Based on the child's zip code at their first event. MSA = metropolitan statistical area (urban area).

### Sensitivity analyses

These analyses were repeated in the subgroup of children who maintained continuous enrollment in PFK from their birth until the end of 2011 (n = 891, 65.5%). The results were similar, with the addition of injuries due to a fall now becoming a significant independent predictor of an increased risk of suspected recurrent NAT events in multivariable modeling (HR 1.42, 95% CI 1.07-1.88, p = 0.02). In order to account for potential selection bias due to informative censoring caused by the removal of children from their home after abuse, the analyses were repeated in only those children followed for at least 60 days after their first event. All results were similar. Finally, all analyses were also repeated in those children who did not have birth records in the PFK database. Compared to children with birth records in PFK, children without birth records tended to be older and to live in a zip code with a slightly higher median family income at their first event documented in PFK (Table 3). Because of these differences and the likelihood that suspected NAT events were missed in children who entered into PFK after birth, these children were not included in the main analyses. There were several differences in the final multivariable models between those with and without birth records in PFK. Specifically, in addition to the risk factors previously identified, children who had a musculoskeletal disease (HR 1.42, 95% CI 1.03-1.92, p = 0.03) or a congenital anomaly (HR 1.44, 95% CI 1.04-2.00, p = 0.03) were more likely to experience a subsequent event.

**Table 3:** Differences between children with and without birth records in PFK

	**Children with birth records in PFK database (N = 1361)**	**Children without birth records in PFK database (N = 898)**	**P**
**Characteristic**			
Male	699 (51.4)	448 (49.9)	0.49
Lives in urban area*	1026 (75.8)	689 (77.0)	0.53
Musculoskeletal disease	166 (12.2)	72 (8.0)	**0.002**
Congenital anomaly	272 (20.0)	84 (9.4)	**<.0001**
Person-months at end of study	29 (17, 40)	26 (14, 37)	**0.0002**
Age			
0-6 months	421 (30.9)	120 (13.4)	**<.0001**
6-12 months	268 (19.7)	131 (14.6)	
12-18 months	248 (18.2)	180 (20.0)	
18-24 months	164 (12.1)	132 (14.7)	
24-30 months	105 (7.7)	113 (12.6)	
> 30 months	155 (11.4)	222 (24.7)	
Enrollment continuity at end of study)			
Continuous enrollment ≥ 24 months	488 (35.9)	302 (33.6)	0.10
Continuous enrollment < 24 months	403 (29.6)	283 (31.5)	
Discontinuous enrollment ≥ 24 months	351 (25.8)	211 (23.5)	
Discontinuous enrollment < 24 months	119 (8.7)	102 (11.4)	
Enrollment breaks over the course of the study			
No break in enrollment	891 (65.5)	585 (65.1)	0.41
One break	331 (24.3)	220 (24.5)	
More than one break (range = 2-5 breaks)	139 (10.2)	93 (10.4)	
Median family income in patient's zipcode*			
$20417-$42043	707 (52.0)	413 (46.0)	**0.006**
$42297-$94873	654 (48.0)	485 (54.0)	
Percent of adults with a bachelor's degree or higher in patient's zipcode*			
0.0-14.0%	686 (50.4)	405 (45.1)	**0.01**
14.2-62.2%	675 (49.6)	493 (54.9)	
Vaccination provided	106 (7.8)	109 (12.1)	**0.0006**
Injury type			
Fracture	315 (23.1)	176 (19.6)	**0.046**
Dislocation	34 (2.5)	32 (3.6)	0.14
Burn	70 (5.1)	47 (5.2)	0.92
Retinal hemorrhage	41 (3.0)	23 (2.6)	0.53
Intracranial	87 (6.4)	41 (4.6)	0.07
Abdominal thoracic	21 (1.5)	6 (0.7)	0.06
Open wound	104 (7.6)	75 (8.4)	0.54
Blood vessel	5 (0.4)	6 (0.7)	0.31
Superficial Injury	114 (8.4)	64 (7.1)	0.28
Contusions	269 (19.8)	163 (18.2)	0.34
Crush	3 (0.2)	2 (0.2)	1
Spinal cord	7 (0.5)	6 (0.7)	0.78
Location of injury			
Head/neck	275 (20.2)	136 (15.1)	**0.002**
Face	64 (4.7)	32 (3.6)	0.19
Chest	64 (4.7)	30 (3.3)	0.11
Abdomen and pelvic contents	49 (3.6)	24 (2.7)	0.22
Extremities or pelvic girdle	337 (24.8)	231 (25.7)	0.61
External	548 (40.3)	347 (38.6)	0.44
Mechanism of injury			
Cut/pierce	6 (0.4)	5 (0.6)	0.7
Drowning/submersion	2 (0.2)	1 (0.1)	1
Fall	223 (16.4)	138 (15.4)	0.52
Fire/Burn	47 (3.5)	28 (3.1)	0.66
Natural/environmental	12 (0.9)	4 (0.5)	0.23
Overexertion	1 (0.1)	0 (0)	1
Poisoning	17 (1.3)	7 (0.8)	0.29
Struck by/against	63 (4.6)	32 (3.6)	0.22
Suffocation	2 (0.2)	1 (0.1)	1
Number of injuries			
0	473 (34.8)	335 (37.3)	**0.048**
1	297 (21.8)	217 (24.2)	
2	225 (16.5)	143 (15.9)	
3	94 (6.9)	69 (7.7)	
4	75 (5.5)	42 (4.7)	
≥5	197 (14.5)	92 (10.2)	
Injury Severity (TMPM-ICD9 probability of death)**	0.031 (0.011, 0.095)	0.021 (0.009, 0.082)	**<.0001**

Characteristics shown were determined at the first event. *Based on the child's zip code at their first event and on data from the 2000 US Census. **TMPM-ICD9 = trauma mortality prediction model. Data are shown as frequencies and percentages for categorical variables and medians and 25^th^ and 75^th^ percentiles for continuous variables. PFK = Partners for Kids Medicaid accountable care organization.

## Discussion

Many children who are victims of NAT may not experience it as a single isolated event, but rather as part of a pattern of recurrent violence that represents the normative structure of their social environment. This study used administrative claims data from a pediatric Medicaid accountable care organization to identify children with repeated medical encounters for injuries that are suspicious for NAT. We have identified several demographic and injury characteristics that are associated with an increased risk for suspected recurrent NAT events. These include living in a rural area, younger age at an event, fewer injuries at an initial event, and specific injury categories including dislocations, superficial injuries, and open wounds. In addition, suspected recurrent NAT events were often observed months after the initial event and the time to a next event decreased with each subsequent event.

Missing child abuse at initial presentation can lead to significant subsequent morbidity [6,19]. With regards to NAT related to TBI, 30% of children hospitalized with abusive head injuries had a sentinel injury [12]. Data from our group using the Ohio State Trauma Registry suggests that victims of recurrent NAT who are hospitalized for their injuries have higher mortality rates compared to victims of single episodes of NAT (25% vs. 10%) [8]. By gaining a better understanding of the types and timing of injuries that portend risk to a child for recurrent NAT, we may be able to develop targeted screening tools and appropriate interventions that can be used to prevent recurrent NAT and its associated morbidity and mortality.

Previously identified risk factors for recurrent NAT include prior child protective services involvement, chronicity of maltreatment, child’s age, and parental history including domestic violence, substance abuse, criminal record, mental health issues, and being maltreated as a child [9-11]. In addition, several case series have described recurrence of maltreatment following specific injuries [5,6,12,13]. On a population level, Friedlaender et al., used Medicaid claims data to demonstrate that victims of maltreatment changed ambulatory care providers with greater frequency in the year before their first episode than those children who were not abused [20]. The current study is the first to utilize system-level administrative data to identify patterns of injuries and factors associated with suspected episodes of recurrent NAT. This population-based approach allows us to examine all medical encounters for a patient, including episodes of care that occur outside the patient’s usual hospital or health care system. Using this approach, we identified several trauma-related risk factors for suspected recurrent NAT.

In this study, more than a quarter of children had a recurrent event within just one year of their first event. Risk factors a recurrent event included having fewer injuries (≤2 injuries) or having a dislocation, open wound, or and superficial injury at the previous event. These data potentially identify a bias in either the diagnosis of abuse and/or the variable response of child protective services to children based on the number and severity of physically evident injuries. Children with fewer or less severe injuries may not be reported to child protective services or are not removed from the unsafe environment leading to subsequent events. Identification of these more minor injuries as potential targets for additional screening or referral to child abuse specialists warrants further prospective study.

This study also found that the median length of time between the first and second suspected NAT events was 191 days (IQR 69, 389). This is important to note because the average length of child protective services involvement with a family may be significantly shorter. In addition, the risk of having a subsequent event increased with each event; 26% of children who experienced a first event proceeded to experience a second event within a year, whereas 60% children who experienced a 4th event proceeded to experience a fifth event within a year. Understanding both the prolonged length of time between a first and second event, as well as the increasing risks with recurrent events may inform secondary prevention strategies for both medical and child welfare staff.

Several limitations inherent in using system-level administrative claims data are relevant to this study. First, approximately 35% of patients had at least one break in Medicaid enrollment. In our analysis, we included these children as if they had remained continuously in the cohort throughout the study period. With this approach, there is the potential that children suffered a recurrent event during the time of non-enrollment, and therefore our data would be an underestimate of the number of recurrent events. However, the appropriateness of this assumption is increased by the finding of similar results in the subgroup of children with continuous enrollment in PFK from birth until the end of the study period. Second, some children who were removed from their home by child welfare after their initial event were lost to follow-up in this study. Whether or not a child remains in PFK after out-of-home placement varies by county in Ohio. Thus, we are unsure of the exact number of children lost to follow-up for this reason. However, when analyses were repeated in children who remained in PFK for at least 2 months after their first event, the results were very similar. Third, we are limited in the sensitivity and specificity of the ICD-9 coding practices used to identify key variables. In particular, ICD-9 coding performed after discharge is likely to underestimate the actual prevalence of abusive injuries in part because physicians may be reticent to assign intentional causality without confirmation from a multi-disciplinary team of social workers and law enforcement agents whose consensus is not often available until after discharge. In addition, ICD-9 codes provide limited ability to distinguish between different types of abuse. In this study, we aimed to focus on suspected physical abuse, but some of the codes chosen to define abuse could have certainly represented instances of emotional or sexual abuse, or child neglect. Furthermore, we were fairly liberal in our definition of potentially abusive injuries. Although some of the injury-only events could have involved accidental injuries, it is important to note that the rate of injury-only episodes was remarkably high in this population (177 events per 1000 person-years), a rate more than 40 times the rate of 3.17 events per 1000 person-years that was reported in a general population of 0–3 year olds [21]. This extraordinarily high injury rate is concerning, regardless of whether the injuries were purposefully inflicted or represent neglect. An additional limitation of this study is that, administrative datasets provide limited data on covariates of interest. For example, this study would have benefited from additional data on race, parental characteristics, and family-level SES characteristics. By integrating US Census data, however, it was possible to evaluate zip-code level SES characteristics. Although the above limitations were unavoidable in the use of this administrative database, it is likely that they mainly resulted in under-identification of suspected NAT events and therefore minimized, rather than exaggerated our findings.

## Conclusion

Factors associated with an increased risk for suspected recurrent NAT events in this study include rural residence, younger age, fewer initially detected injuries, and specific injury types at a previous event. Recurrent events often occur months after the initial event. These findings potentially identify a bias in either the diagnosis of NAT or the response of child protective services to children who present with less severe or less numerous injuries.

## Abbreviations

NAT: Non-accidental trauma; PFK: Partners for kids; ICD-9-CM: International classification of diseases, ninth revision, clinical modification; CPT: Current procedural terminology; TMPM-ICD9: Trauma mortality prediction model; SES: Socioeconomic status; PWP-GT: Prentice, Williams and Peterson gap time model; HR: Hazard ratio.

## Competing interests

The authors declare that they have no competing interests.

## Authors’ contributions

KJD conceptualized and designed the study, interpreted the data, drafted parts of the initial manuscript, reviewed and revised the manuscript, and approved the final manuscript as submitted. JT interpreted the data, drafted parts of the initial manuscript, reviewed and revised the manuscript, and approved the final manuscript as submitted. JIG interpreted the data, critically reviewed and revised the manuscript, and approved the final manuscript as submitted. JNC acquired and analyzed the data, interpreted the data, drafted parts of the initial manuscript, reviewed and revised the manuscript, and approved the final manuscript as submitted. PCM conceptualized and designed the study, interpreted the data, drafted parts of the initial manuscript, reviewed and revised the manuscript, and approved the final manuscript as submitted. All authors read and approved the final manuscript.

## Pre-publication history

The pre-publication history for this paper can be accessed here:

http://www.biomedcentral.com/1471-2431/14/217/prepub

## Supplementary Material

Additional file 1 **STROBE Statement—Checklist of items that should be included in reports of *cohort studies*.** Click here for file
